# Significance of computed tomography combined with postural stimulation test in predicting laterality of primary aldosteronism

**DOI:** 10.1186/s12902-023-01281-x

**Published:** 2023-02-03

**Authors:** Yingxing Wu, Zuxiang Wu, Huan Hu, Jingan Rao, Chenkai Hu, Qiang Peng, Ping Li

**Affiliations:** Department of cardiovascular medicine, The second affiliated hospital of Nanchang University, Nanchang, 330006 Jiangxi China

**Keywords:** Primary aldosteronism, Laterality, Adrenal CT, Postural stimulation test, Adrenal venous sampling

## Abstract

**Backgrounds:**

Adrenal venous sampling (AVS) represents the gold standard for classifying primary aldosteronism (PA). However, AVS is a technically demanding, expensive and invasive procedure. Computed tomography (CT) scans is recommended as the initial study of classification diagnosis by the current guidelines. In addition, postural stimulation test (PST) has been used to provide additional subtype diagnostic information.

**Objective:**

This work aimed to evaluate the diagnostic utility of the adrenal CT combined with PST in the classification diagnosis of PA.

**Methods:**

We analyzed PA patients who underwent AVS from November 2017 to February 2022 at a single center. Subtype classification of PA was determined by AVS. We analyzed the concordance rate between AVS outcomes, adrenal CT, and PST, and explored the value of adrenal CT combined with PST for predicting laterality of PA.

**Results:**

Total 531 PA patients were included in the present study. The concordance rate between AVS and the adrenal CT was 51.0%(271/531). Receiver operating characteristic (ROC) curve of PST showed that the area under curve (AUC) was 0.604 [95% confidence interval (CI): 0.556, 0.652], the optimal cut-off value was 30%. The sensitivity, specificity, positive predictive value (PPV), negative predictive value (NPV), positive likelihood ratio (+LR), and negative likelihood ratio (−LR) of PST for diagnosis bilateral PA on AVS was 72.8, 46.2%, 0.48, 0.71, 1.35, and 0.59, respectively. The prevalence of unilateral PA on AVS in patients with unilateral lesion on CT and negative PST, unilateral lesion on CT and positive PST, bilateral normal or lesions on CT and negative PST, and bilateral normal or lesions on CT and positive PST was 82.4% (108/131), 59.9% (91/152), 50.7% (37/73), and 44.6% (78/175), respectively. The sensitivity, specificity, PPV, NPV, +LR, and -LR of adrenal CT combined with PST for the diagnosis of unilateral PA were 34.4, 89.4%, 0.82, 0.49, 3.25, and 0.73, respectively.

**Conclusions:**

The combination of CT findings and PST can improve the accuracy of predicting laterality of PA.

**Supplementary Information:**

The online version contains supplementary material available at 10.1186/s12902-023-01281-x.

## Introduction

Primary aldosteronism (PA) is the most common form of secondary hypertension and is largely unrecognized [1]. Compared with essential hypertensives, PA patients have a higher rate of cardiovascular complications and target organ damage due to the excessive secretion of aldosterone through the adrenal cortex [2–4]. Aldosterone-producing adenomas (APA) and idiopathic hyperaldosteronism (IHA) are the most common forms of PA, accounting for 35 and 60% of all cases, respectively [5]. Patients with APA can be cured by unilateral adrenalectomy, while patients with IHA require lifelong treatment with mineralocorticoid receptor (MR) antagonists [6]. Although adrenal venous sampling (AVS) is recommended as a gold standard for classifying unilateral and bilateral subtypes, this approach has a risk of adrenal vein rupture and thromboembolism, successful cannulation largely depended on the radiologist’s experience and skills, and a lack of standardization in both the procedure and the interpretation [7–10]. Therefore, a simpler non-invasive method to predicting laterality without performing AVS is needed.

The current guideline recommends adrenal computed tomography (CT) scans as the initial study for the subtype diagnosis of PA and to exclude adrenocortical carcinoma [6]. Adrenal CT cannot distinguish non-functioning incidentalomas and determine the presence of microadenoma, thus the subtype classification based on adrenal CT findings does not have sufficient accuracy as compared with AVS [11–13]. Many studies have demonstrated that adrenal CT findings combined with some factors, including serum potassium, plasma aldosterone concentration (PAC), and the results of confirmatory tests, can accurately predict PA subtype [14–17]. That is, additional indicators can provide valuable information on the laterality of adrenal lesions.

Postural stimulation test (PST) has been shown to be useful for predicting PA subtype based on aldosterone changes post-upright posture [18, 19], but there are few relevant studies with conflicting results. A recent study showed an 85.0% accuracy rate for the diagnosis of APA by AVS and/or postoperative pathology in patients with unilateral nodules on CT and no response to PST [20]. However, the value of adrenal CT combined with PST in predicting laterality PA is still unknown.

The aim of present study was to evaluate the diagnostic utility of the adrenal CT combined with PST in the classification diagnosis of PA.

## Methods

### Patient cohort

In this retrospective study we recruited 531 PA patients with available data of clinical characteristics, biochemistry indexes, results of detection and confirmatory tests, CT findings, outcomes of PST, and successful AVS between November 2017 and February 2022 by searching the electronic information system of the second affiliated hospital of Nanchang university. The study was performed in accordance with the Declaration of Helsinki and approved by The Medical Research Ethics Committee of The Second Affiliate Hospital of Nanchang University. Written informed consent was obtained from all patients.

### Diagnosis of PA

All patients were diagnosed with PA according to the Endocrine Society Clinical Practice Guidelines [6] and the Expert Consensus on the Diagnosis and Treatment of Primary Aldosteronism in China [21]. Before testing, treatment with diuretics and MR antagonists has been discontinued > 6 weeks and treatment with beta-adrenergic receptor blockers, dihydropyridine calcium channel blockers, angiotensin II receptor blockers and angiotensin-converting enzyme inhibitors was discontinued > 2 weeks before screening test. Antihypertensive medications were usually replaced with a non-dihydropyridine calcium channel blockers, alpha-adrenergic receptor blockers or both, and BP monitoring was performed. Patients were required to preserve normal sodium intake and to maintain serum potassium to normal or near normal status. The positive screening test was performed a ratio of PAC (measured in ng/dL) to plasma renin activity (PRA) (measured in ng/mL/h) (aldosterone to renin ratio, ARR) > 30 and PAC > 15 ng/dL. Blood samples were obtained after 2 hours in the upright position. Confirmatory tests for PA included the captopril challenge test (CCT) and saline infusion test (SIT). If the aldosterone suppression rate was < 30% or PAC post-CCT was > 11 ng/dl after 2 hours 50 mg of captopril orally, CCT was considered positive for PA. The PAC > 10 ng/dL after 4 hours continuous intravenous administration of 2 L of 0.9% saline on seated position could be considered as the criterion of SIT positive.

### Postural stimulation test

PST was performed in a controlled inpatient setting. The first blood samples were collected at 05:00 a.m. on the following day in the supine position, and a second one after 2 hours in the upright position. PAC and PRA were quantified in both samples as soon as possible.

### CT imaging

All patients underwent thin-slice adrenal CT before AVS, and therefore the radiological evaluation of CT was performed under clinical diagnosis such as PA or suspected PA but in the absence of AVS data. Meanwhile, this routine can assist the interventional radiologist in obtaining anatomy information to improve success rates. The results were evaluated by radiologists and classified into three categories: unilateral lesion, bilateral lesion, and bilateral normal. Adrenal lesion of CT was defined as adenoma, nodule or hyperplasia if adrenal gland thickness measured > 10 mm in diameter. Bilateral normal appearance was defined if the thickness of adrenal gland was < 10 mm on both sides.

### AVS protocol and criteria

The subtype of PA was determined based on the outcomes of simultaneous AVS without adrenocorticotropic hormone (ACTH) stimulation. Blood samples were collected from both adrenal veins and the inferior vena cava below the renal veins. Successful cannulation of the adrenal vein was defined as a selectivity index (SI) > 2, calculated as the ratio of cortisol concentration in the adrenal vein to that in the inferior vena cava. To determine the PA subtype, we used the lateralization index (LI), which was calculated by dividing aldosterone to cortisol ratio on the dominant side by that on the non-dominant side. Patients with unilateral PA had a LI of > 2, while those with bilateral PA had a LI of < 2 [6, 10, 21].

### Assay methods

PAC and PRA were determined by Chemiluminescence immunoassay (CLIA) using commercially available kits. CLIA kits and detecting instrument (Sinbe Co., LTD, Shenzhen, China) were used to detected aldosterone and renin concentrations in plasma as the manufacturer’s instruction. The reference range of PAC in the supine position was 30 to 160 pg/mL and 70 to 300 pg/mL in the upright position. The intra-assay coefficient of variance (CV) of aldosterone was ≤10%, and the inter-assay CV was ≤5% at concentrations of 5–1000 pg/mL. Meanwhile, The reference range of PRA in the supine position was 0.15 to 2.33 ng/mL/h and 0.10 to 6.56 ng/mL/h in the upright position. The intra-assay CV of angiotensin I was ≤15%, and the inter-assay CV was ≤10% at concentrations of 0.1–24.0 ng/mL. Plasma cortisol concentration (PCC) were determined with the ADVIA Centaur XP by CLIA (Siemens Co., German). The reference range of PCC in the 7–9 am was 5.27 to 22.45 μg/dL. The intra-assay CV of PCC was ≤10%, and the inter-assay CV was ≤7% at concentrations of 0.5–75 μg/dL.

### Statistical analysis

SPSS (version23.0; SPSS Inc., Chicago, IL, USA) was used in the whole statistical analysis. All quantitative normally distributed variables are reported as means with SDs and quantitative non-normally distributed variables are presented as medians with IQRs. Categorical variables are presented as absolute numbers and percentages. We analyzed quantitative normally distributed variables using unpaired *t*-test. We analyzed group differences using Kruskal-Wallis or Mann-Whitney *U* tests for quantitative non-normally distributed variables, and *χ*^*2*^ or Fisher’s exact tests for categorical variables. Clinical characteristics and biochemistry indexes were compared among patients with unilateral and bilateral PA. Receiver operating characteristics (ROC) curves were used to establish an optimized aldosterone change rate threshold to distinguish between unilateral and bilateral PA. CT findings (unilateral lesion, bilateral lesion and bilateral normal on CT), outcomes of PST (positive and negative), and their combination were investigated in the subtype diagnosis of PA determined by AVS. *P* <0.05 (two-tailed) was defined as statistically significant difference.

## Results

### Patient cohort

A total of 531 PA patients with successful AVS were included in this study (average age 48.99 ± 9.96 years; 53.86% males). In our study, the prevalence of hypokalemia (serum potassium level of < 3.5 mmol/L) and unilateral PA were 43.31%(230/531) and 59.1%(314/531), respectively. The distributions of study participant baseline characteristics according to the classification of AVS outcomes are presented in Table 1. Patients with unilateral PA had higher values for systolic blood pressure, duration of hypertension, and serum sodium level, and lower values for serum potassium level, compared with bilateral PA (all *P* <0.05) (Table 1).

**Table 1:** Comparison of baseline characteristics between unilateral and bilateral PA

	Total (*n* = 531)	Unilateral PA (*n* = 314)	Bilateral PA (*n* = 217)	*P* value
Age (y)	48.99 ± 9.96	48.72 ± 10.00	49.39 ± 9.89	0.445
Sex [male (%)]	286(53.86)	175(55.73)	111(51.15)	0.298
BMI (kg/m^2^)	25.67 ± 3.44	25.65 ± 3.42	25.69 ± 3.49	0.887
Diabetes [n (%)]	53(9.98)	34(10.83)	19(8.76)	0.434
SBP (mm Hg)	155.88 ± 20.25	157.68 ± 20.72	153.27 ± 19.32	0.014
DBP (mm Hg)	94.84 ± 14.60	95.75 ± 14.54	93.53 ± 14.63	0.084
Duration of hypertension (y)	4(0.5,10)	5(1,10)	3(0.1,8.5)	0.001
TC (mmol/L)	4.70 ± 1.02	4.66 ± 1.00	4.77 ± 1.06	0.239
TG (mmol/L)	1.48(1.04,2.13)	1.48(1.00,2.16)	1.48(1.07,2.15)	0.741
HDL-c (mmol/L)	1.15 ± 0.34	1.16 ± 0.34	1.14 ± 0.32	0.638
LDL-c (mmol/L)	2.71 ± 0.84	2.68 ± 0.80	2.74 ± 0.89	0.413
Hcy (mmol/L)	11.88(9.53,14.22)	11.98(9.67,14.33)	11.70(9.36,14.09)	0.340
FBG (mmol/L)	5.34 ± 1.32	5.36 ± 1.25	5.31 ± 1.43	0.626
HbA1c (%)	5.54 ± 0.69	5.54 ± 0.70	5.54 ± 0.68	0.969
eGFR (mL/min/1.73 m^2^)	98.22 ± 22.40	99.17 ± 23.85	96.84 ± 20.09	0.224
Cr (μmol/L)	72.94 ± 17.80	72.85 ± 17.68	73.06 ± 18.02	0.898
UA (μmol/L)	357.10 ± 90.79	355.53 ± 89.39	359.37 ± 92.94	0.633
Serum potassium (mmol/L)	3.55 ± 0.50	3.41 ± 0.51	3.75 ± 0.41	< 0.001
Serum sodium (mmol/L)	141.37 ± 2.28	141.60 ± 2.24	141.02 ± 2.29	0.004

### Diagnostic concordance rate between CT finding and AVS

The proportion of bilateral normal, bilateral lesion, and unilateral lesion on CT findings was 32.8% (174/531), 13.9% (74/531), and 53.3% (283/531), respectively. The total prevalence of unilateral hyperaldosteronism on AVS was 59.1% (314/531). Prevalence of unilateral PA on AVS was higher in patients with unilateral lesion on CT (70.3%, 199/283) than those with bilateral normal on CT (40.2%, 70/174, *P* < 0.001), but not higher than those with bilateral lesion on CT (60.8%, 45/74, *P* = 0.117). Although CT finding were significantly associated with the subtype diagnosis of PA by AVS outcome, the total diagnostic concordance rate of CT findings was 51.0% (271/531). About 40.2% (70/174) of patients with bilateral normal on CT showed unilateral hyperaldosteronism on AVS. Furthermore, 61 patients (21.6%, 61/283) with unilateral lesion on CT showing discordant lateralization on AVS to contralateral adrenal gland (Table 2, supplementary Fig. 1). The diagnostic results of AVS and CT finding were in poor agreement (Kappa = 0.241, *P* < 0.001).

**Table 2:** Diagnostic concordance rate between CT finding and AVS

CT finding	Outcomes of AVS			Concordance of CT finding,%(n/N)	Prevalence of Unilateral PA on AVS,%(n/N)
	Bilateral	Unilateral			
		Left	Right		
Bilateral(*n* = 248)					
Normal(*n* = 174)	104	17	53	59.8(104/174)	40.2(70/174)
Lesion(*n* = 74)	29	20	25	39.2(29/74)	60.8(45/74)
Unilateral(*n* = 283)					
Left (n = 217)	73	89	55	48.8(138/283)	70.3(199/283)
Right(*n* = 66)	11	6	49		
Total				51.0(271/531)	59.1(314/531)

### PRA, PAC, and ARR during postural stimulation test

Overall, patients with both bilateral PA and unilateral PA had significantly higher PRA and PAC after holding an upright position for 2 hours. There was no significant difference in supine PRA between unilateral PA and bilateral PA, but upright PRA was lower in patients with unilateral PA (*P* <0.05). Patients with unilateral PA presented a higher aldosterone level both at baseline and upright position, but show a lower PAC changes. In addition, patients with unilateral PA showed a higher ARR levels in both supine and upright position compared with bilateral PA (all *P* < 0.05) (Table 3).

**Table 3:** Comparison of PRA, PAC, and ARR during postural stimulation test between unilateral and bilateral PA on AVS

	Posture	Unilateral PA (*n* = 314)	Bilateral PA (*n* = 217)	*P* value
PRA [ng/(ml*h)]	Supine	0.01(0.01,0.10)	0.02(0.01,0.16)	0.063
	Upright	0.11(0.02,0.41)	0.19(0.04,0.48)	0.003
	Δ	0.04(0.01,0.28)	0.12(0.01,0.33)	0.008
PAC (ng/dl)	Supine	19.4(14.0,31.1)	15.7(12.0,20.2)	< 0.001
	Upright	26.6(21.3,38.8)	23.5(18.9,30.3)	< 0.001
	Δ	6.3(2.5,12.1)	7.9(4.8,11.0)	0.023
ARR (ng/dl)/[ng/(ml*h)]	Supine	1102(173,2186)	621(105,1510)	< 0.001
	Upright	323(63,1563)	111(51,564)	< 0.001

### Diagnostic accuracy of postural stimulation test

**Fig. 1:**
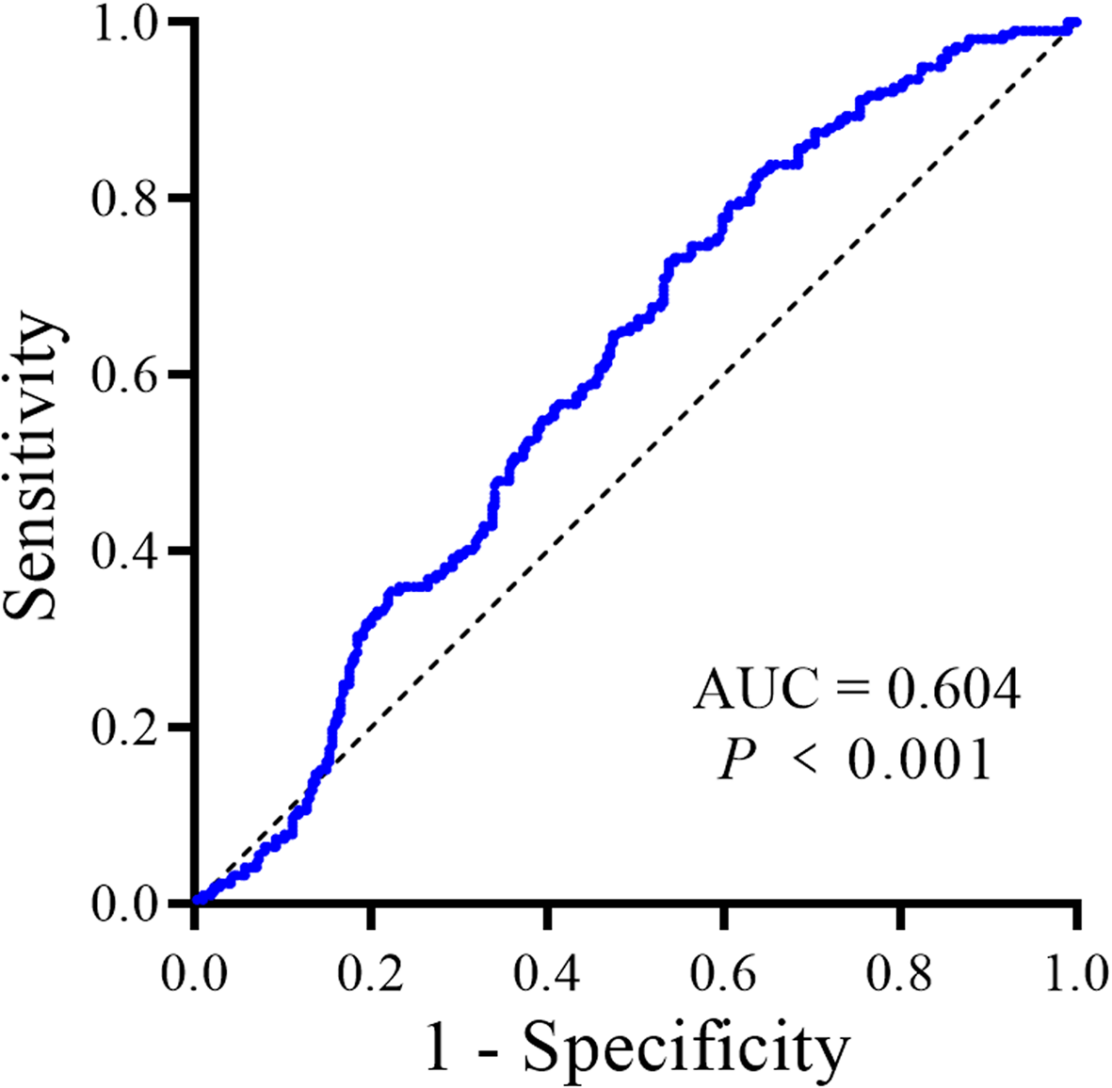
ROC curve analysis of aldosterone change rate to diagnose bilateral subtype

According to the ROC analysis, the optimized cut-off value was a 30% increase in aldosterone (area under curve (AUC) = 0.604; 95% confidence interval (CI): 0.556–0.652; *P* < 0.001) (Fig. 1). By using this cut-off, PST had a sensitivity of 72.8%, specificity of 46.2%, positive predictive value (PPV) of 0.48, negative predictive value (NPV) of 0.71, positive likelihood ratio (+LR) of 1.35, and negative likelihood ratio (−LR) of 0.59 for prediction bilateral PA. In our cohort, 158 out of 217 patients with bilateral PA showed a positive PST (defined by increase in aldosterone of ≥30%). When using the cut-off of an increase in aldosterone of 127% or more after postural stimulation, PST showed a specificity of 90.1% for prediction bilateral PA (supplementary Tables 1, 2). Prevalence of bilateral PA on AVS was higher in patients with positive in PST (48.3%, 158/327) than those with negative PST (28.9%, 59/204, *P* < 0.001).

### Prevalence of unilateral PA on AVS predicted by combination of CT findings and postural stimulation test

The prevalence of unilateral PA was investigated by combination of CT findings and PST. The prevalence of unilateral PA on AVS was 82.4% (108/131) in patients with unilateral lesion on CT and negative in PST, whereas, it was 59.9% (91/152) in patients with unilateral lesion on CT and positive in PST. In patients with bilateral normal or lesions on CT, the prevalence of unilateral PA on AVS was 50.7% (37/73) in patients with negative PST, and 44.6% (78/175) in patients with positive PST outcomes. The sensitivity, specificity, PPV, NPV, +LR, and -LR of adrenal CT combined with PST for the diagnosis of unilateral PA were 34.4, 89.4%, 0.82, 0.49, 3.25, and 0.73, respectively (Table 4).

**Table 4:** Prevalence of unilateral PA on AVS by categorizing the combination of CT findings and PST

Outcomes		Unilateral PA (*n* = 314)	Bilateral PA (*n* = 217)	Prevalence of Unilateral PA on AVS,%(n/N)
CT^a^	PST^b^			
+	–	108	23	82.4(108/131)
+	+	91	61	59.9(91/152)
–	–	37	36	50.7(37/73)
–	+	78	97	44.6(78/175)
Total		314	217	59.1(314/531)

^a^“+”:unilateral lesion (including adenoma, nodule or hyperplasia) on adrenal CT, “-”:bilateral noraml or lesion on CT

^b^“+”:negative in PST, “-”:negative in PST (defined by increase in aldosterone of ≥30%)

## Discussion

Our study showed that the optimal cut-off value for subtype classification by PST was 30%, and a percentage less than 30% was taken as a negative result (indicating a high likely of unilateral PA). The combination of CT findings and PST can improve the accuracy of predicting laterality of PA and help determine which patients need to undergo AVS for subtype classification.

The relatively higher rate of cardiovascular complications and target organ damage has led clinicians to increasingly recognize the importance of PA diagnosis. Case detection, case confirmation and subtype classification are the three key steps in PA diagnosis [6]. Subtype classification of PA is essential for selecting treatment strategies. The optimal treatment for patients with APA is unilateral adrenalectomy, whereas patients with IHA are best treated with MR antagonists [5, 6, 21]. AVS is recommended the gold standard method for assessing lateralization of aldosterone secretion in the current guidelines for PA [6, 21]. However, the shortcomings of AVS are becoming more obvious. First, AVS is an invasive procedure with a risk of adrenal vein rupture, thromboembolism, and periadrenal hematoma [9]. On the other hand, AVS is a technically challenging technique that require the interventional radiologist to have great skills and experience. The success rates not even reaching 50% in some centers [8, 22]. In additional, AVS is an expensive procedure and only available in a small number of specialized hypertension centers [10]. Finally, there are no standards for the procedure and the criteria used to interpret the results [10, 23]. Therefore, it is important to appropriate select PA patients who need AVS.

Our results have shown that the overall concordant rate between CT findings and AVS outcomes was 51.0%, and the concordant rate for unilateral lesion, bilateral lesion and bilateral normal on CT was 48.8, 39.2 and 59.8%, respectively. This result is consistent with previous studies, which report the concordance rate of CT with AVS subtyping to be 53–68% [11–13]. In one study of 203 patients with PA, 42 patients (22%) would have been incorrectly excluded as candidates for adrenalectomy, and 48 (25%) might have had unnecessary or inappropriate surgery based on CT findings alone [11]. It’s indicated that AVS is crucial to the choice of treatment options. However, a randomized controlled trial showed that treatment of PA based on CT or AVS did not show significant differences in intensity of antihypertensive medication or clinical benefits for patients after 1 year of follow-up [24]. This finding plausibly challenges the current recommendation of AVS, but long-term clinical effects of managements based on CT findings may require further observation. Although CT is not accurate enough for subtype classification, CT finding in combined with other clinical variables can be applied to predict which PA patients should spare AVS [14–17].

The value of PAC change after holding an upright position for 2 hours in unilateral PA group was significantly lower than that in bilateral PA group, suggesting that the intensity of response to postural stimulation varied between two subtypes. In our study, 158 patients (48.3%) with positive in PST were diagnosed bilateral PA by AVS. In contrast, only 28.9% of patients with negative in PST were diagnosed bilateral PA by AVS. Our study showed that the optimal cut-off value for subtype classification by PST was 30% according to the ROC curve, and the PST was associated with the subtype diagnosis on AVS. A recent study found that showed a specificity of 100% at a sensitivity of 36.4% for prediction of unilateral PA by using cut-off value of a 28% decrease in aldosterone after 4 hours of upright [19]. In an analysis of 50 PA patients, Lau et al [25] suggested poor diagnostic accuracy for differentiation between APA and bilateral adrenal hyperplasia (BAH) using a fall in aldosterone of ≥30% as evidence of APA and a rise of ≥30% as suggestive of BAH. These conflicting results might be attributed to the differences in the methodology of the PST and the wide variation of study populations in different regions.

We then evaluate the diagnostic utility of the adrenal CT combined with PST in the classification diagnosis of PA. The prevalence of unilateral PA on AVS was highest in patients with unilateral lesion on CT and negative in PST followed by unilateral lesion on CT with positive in PST and bilateral normal or lesion on CT with negative in PST. The current guideline suggests that younger patients (age below 35) with spontaneous hypokalemia, marked aldosterone excess, and unilateral adrenal lesions on adrenal CT scan may not need AVS before proceeding to unilateral adrenalectomy [6]. Umakoshi et al [13] proposed that the combination of CT findings and potassium status does not replace the essential role of AVS in subtype diagnosis, but it could provide evidence for a graded recommendation for it according to the prevalence of unilateral hyperaldosteronism on AVS. Our results indicated that patients with unilateral lesion on CT and negative in PST have a high probability of a lateralized form of AVS and should be strongly recommended to undergo AVS to distinguish PA subtypes. In addition, the combination of CT finding and PST can provide assistance information when AVS is unsuccessful. We expect to provide objective evidence for subtype diagnosis for some institutions that are unable to conduct AVS, while reducing the number of PA patients requiring AVS.

## Limitation

Our study have several limitations. First, our study was a single-center, observational, retrospective study. A prospective study is therefore required to confirm and validate the accuracy of the present outcomes. However, our study had a greater number of patients than most previous studies. Second, in this study, only patients who had successful AVS were eventually included. Therefore, the selection biases may exist in the present study, which may limit its validity in actual clinical practice. Third, the subtype diagnosis of PA in this study was based on AVS outcomes, with the false-positives and false-negative results. We need to further adjust the patient grouping according to the pathological outcome and the prognosis.

## Conclusions

Our results indicated that the combination of CT findings and PST can improve the accuracy of predicting laterality of PA. AVS is strongly recommended for patients with unilateral lesion on CT and negative PST before surgery by showing a high likelihood of a lateralized form on AVS.

## Supplementary Information

**Additional file 1: Table 1.** Characteristic of ROC analysis for the ability of the aldostrone change rate to diagnose bilateral PA. **Table 2.** Sensitivity and specificity of different cut-off value of PST for detection of bilateral PA. **Figure 1.** outcomes of AVS for the different CT findings.

## Data Availability

All data relevant to this study are available from the corresponding authors upon reasonable request.
