# Supplementary material for: Significance of computed tomography combined with postural stimulation test in predicting laterality of primary aldosteronism
Source: BMC Endocr Disord. 2023 Feb 3;23:32. doi: 10.1186/s12902-023-01281-x (PMC9898977; doi:10.1186/s12902-023-01281-x)
Supplement: Supplementary file 1 — Additional file 1: Table 1. Characteristic of ROC analysis for the ability of the aldostrone change rate to diagnose bilateral PA. Table 2. Sensitivity and specificity of different cut-off value of PST for detection of bilateral PA. Figure 1. outcomes of AVS for the different CT findings. [file 12902_2023_1281_MOESM1_ESM.docx]

**Supplementary Material**

Table 1. Characteristic of ROC analysis for the ability of the aldostrone change rate to diagnose bilateral PA

| AUC | 95%CI | Best cut-off value | Sensitivity | Specificity | PPV | NPV | +LR | -LR |
| --- | --- | --- | --- | --- | --- | --- | --- | --- |
| 0.604 | 0.556-0.652 | 30.00% | 72.8% | 46.2% | 0.48% | 0.71% | 1.35 | 0.59 |

AUC. area under the curve, CI. confidential interval, PPV. positive predictive value, NPV. negative predictive value, +LR. positive likelihood ratio, -LR. negative likelihood ratio.

Table 2. Sensitivity and specificity of different cut-off value of PST for detection of bilateral PA

| cut-off value | Sensitivity  (%) | Specificity  (%) | +LR | -LR | TP  (n) | FP  (n) | TN  (n) | FN  (n) | PPV  (%) | NPV  (%) |
| --- | --- | --- | --- | --- | --- | --- | --- | --- | --- | --- |
| 127% | 7.4 | 90.1 | 0.75 | 1.03 | 16 | 31 | 283 | 201 | 0.34 | 0.58 |
| 100% | 16.1 | 84.7 | 1.06 | 0.99 | 35 | 48 | 266 | 182 | 0.42 | 0.59 |
| 80% | 30.9 | 81.0 | 1.62 | 0.85 | 67 | 60 | 254 | 150 | 0.53 | 0.63 |
| 50% | 52.5 | 61.5 | 1.36 | 0.77 | 114 | 121 | 193 | 103 | 0.49 | 0.65 |
| 30% | 72.8 | 46.2 | 1.35 | 0.59 | 158 | 169 | 145 | 59 | 0.48 | 0.71 |
| 10% | 89.4 | 25.2 | 1.19 | 0.42 | 194 | 235 | 79 | 23 | 0.45 | 0.77 |
| 0% | 94.9 | 15.3 | 1.12 | 0.33 | 206 | 266 | 48 | 11 | 0.44 | 0.81 |

TP. true positives, FP. false positive, TN. true negative, FN. false negative, +LR. positive likelihood ratio, -LR. negative likelihood ratio, PPV. positive predictive value, NPV. negative predictive value.

Figure 1. outcomes of AVS for the different CT findings
